# Online Gambling among Treatment-Seeking Patients in Singapore: A Cross-Sectional Study

**DOI:** 10.3390/ijerph15040832

**Published:** 2018-04-23

**Authors:** Melvyn Zhang, Yi Yang, Song Guo, Chris Cheok, Kim Eng Wong, Gomathinayagam Kandasami

**Affiliations:** National Addictions Management Service (NAMS), Institute of Mental Health, 10 Buangkok Green Medical Park, Singapore 539747, Singapore; yi_yang@imh.com.sg (Y.Y.); Song_guo@imh.com.sg (S.G.); Chris_cheok@imh.com.sg (C.C.); kim_eng_wong@imh.com.sg (K.E.W.); gomathinayagam_kandasami@imh.com.sg (G.K.)

**Keywords:** gambling disorder, online gambling, addiction, Singapore, epidemiology

## Abstract

Given that technology has greatly facilitated easier access to gambling in previous years, it is timely to look in-depth into online gambling activities and behaviors. There have been several studies that examined online gambling. However, most of the current studies to date have focused on determining the prevalence and the epidemiology of problem gambling arising from online gambling in Western cohorts. There remains a paucity of research looking at the problem of online gambling among Asian individuals. The objectives of the current study are to elucidate the characteristics of online gambling among an Asian cohort and to explore the harm associated with online gambling and the potential mechanisms by which harm associated with online gambling could be minimized. It is hoped that the findings of the current paper will bridge the existing gaps in the research literature. A cross-sectional study design was utilized to recruit 100 participants who were attending outpatient services at the National Addictions Management Service (NAMS) from March 2014 to October 2015. The majority of the participants were male, of Chinese ethnicity and under the age of 30 years old (48%). Mobile phones and smartphones were the most commonly utilized platforms for gambling online. The median largest ever debt incurred as a result of online gambling ($20,000) was significantly more than that due to offline gambling ($500) (*Z* = −4.17, *p* < 0.001). As for the biggest ever loss, participants had incurred a significantly larger median loss from online gambling ($7000) (*Z* = −2.73, *p* < 0.01) compared to offline gambling ($2000). A total of 18.4% of participants had waited between 1 to 2 years from their first online gambling experience to seek treatment and 17.3% had waited for more than 10 years. This is perhaps one of the first Asian studies to investigate the serious harm involved in online gambling. The findings from our study are intended to guide further interventions in the treatment of online gambling related disorders; and would be of interest to governmental organizations in their planning of regulations for online gambling.

## 1. Introduction

Since the introduction of the Diagnostic and Statistical Manual (DSM-5) [1], the term, pathological gambling disorder has been replaced by gambling disorder. Based on the new diagnostic criteria, individuals are required to fulfill a minimum of four out of the nine criteria to be diagnosed with a gambling disorder. The changes in the criteria for diagnosis of gambling disorders might have resulted in more individuals being diagnosed with a gambling disorder. Apart from the recent revision in the diagnostic criteria for gambling, advances in technologies have led to a new method of gambling, that of online gambling. Internet technology provides the opportunity to gamble anywhere and at any time. Prior studies have reported that there has been an increased incidence of problem gambling [2]. Problem gambling has been found to be associated with a myriad of psychiatric disorders, ranging depression and anxiety disorders to substance use disorders [3]. Suicidal tendencies and mortality are increased amongst problem gamblers, particularly amongst those who have comorbid depression [4]. Whilst the changing trends in gambling disorders could be accounted for by the availability of more novel mechanisms through which individuals can gamble, legislative changes are also responsible to some extent.

In an Asian country like Singapore, whilst gambling remains a harmless pastime for most individuals, there are some individuals who experience negative consequences which may then progress to disordered (problem or pathological) gambling, requiring treatment. A follow-up study of the responses of pathological gamblers to treatment at the National Addictions Management Service (NAMS), Singapore showed that there were close to 400 pathological gamblers seen and treated in 2014 [5]. The National Council on Problem Gambling (NCPG) in Singapore has been conducting general population surveys every three years on gambling activities among Singapore residents [6]. Whilst the previous surveys conducted have generally shown relatively low rates of past-year participation in online gambling, the rates of online gambling have been gradually increasing from 0.1% in 2005 to that of 1% in 2008 [6]. The prevalence of online gambling has seemed to stabilize at 1% based on the last survey which was conducted in 2014 [7]. One of the key findings from the previous survey conducted was that online gamblers self-reported that they had experienced more issues pertaining to self-control compared to gamblers who were participating in conventional modalities of gambling. In addition, online gamblers also reported in the survey that they had spent more time gambling than they had planned to, as well as gambled with more money than they had planned for.

Given that technology has greatly facilitated improved and easier access in recent years, it is timely to look in-depth at online gambling activities and behaviors. There have been several studies which have looked at online gambling [8,9]. However, most of the current studies to date have focused on determining the prevalence and epidemiology of problem gambling arising from online gambling. Of note, most of these studies have been conducted in Western cohorts. Given that the current research has clearly demonstrated a myriad of negative consequences associated with conventional mechanisms of gambling, a re-look into online gambling behaviors is required, particularly regarding the new mechanisms of gambling, as well as potentially the consequences that could arise from online gambling and legislative changes. Given the existing gaps in knowledge, the existing study has been conceptualized to explore the online gambling behaviors in a sample of treatment-seeking participants. The specific objectives of the current study are to elucidate the characteristics of online gambling amongst an Asian cohort and to explore the harm associated with online gambling and the potential mechanisms by which the harm associated with online gambling could be minimized.

## 2. Methodology

### 2.1. Ethical Approval and Funding

This research study has been approved by the National Healthcare Group’s Domain Specific Review Board in 2013 (NHG DSRB Reference: 2013/01253). The Ministry of Social and Family Development (MSF) provided the study funding with the aim of exploring the nature and consequential impact of online gambling among treatment-seeking problem online gamblers.

### 2.2. Study Design and Inclusion and Exclusion Criteria

A cross-sectional study design was utilized to recruit 100 participants who were attending outpatient services at the NAMS from March 2014 through to October 2015. For the participants to be considered for the current research study, they had to fulfill the following inclusion criteria: (a) aged 21 to 70 years old, and (b) having gambled online on at least three occasions in the year prior to giving their informed consent. Participants with active symptoms of a major psychiatric disorder such as schizophrenia or bipolar disorder were excluded from the study. Participants took the survey during their attendance to the service in 2014 through to 2015.

### 2.3. Sampling and Sample Size

Cross-sectional convenience sampling was utilized in the recruitment of the subjects. A total sample of 100 treatment-seeking participants, including 98 males and 2 females, aged between 21 and 70 years old, who presented to NAMS between 2014 through to 2015 were recruited.

### 2.4. Recruitment Process

Potential participants who fulfilled the inclusion criteria were referred by the members of the NAMS research team sited at the NAMS outpatient specialist clinic by their psychiatrists. Aside from direct referral, an existing outpatient database was also used to identify the potential participants. Participants who were referred or who were identified to be suitable were then briefed in further detail about the purpose of the current research project, as well as the procedure and the duration. Informed consent was obtained from each participant. The questionnaire survey was administered to the participants in a quiet and private room, to ensure the privacy of the participants and also to minimize any potential distractions. Participants were given a total duration of one hour to complete the necessary questionnaire. Upon completion of the questionnaire, they were reimbursed with an inconvenience fee.

### 2.5. Questionnaires Administered

The following questionnaires, which were in English, were administered to the participants to collate the necessary information for the research study.

The basic demographic information of the participants was collated from a questionnaire. These included age, nationality, level of education, race, marital status, employment and current occupation. In addition, questions were asked to determine whether participants have had a family history of gambling related disorders.

For the gambling characteristics questionnaire, information was collated with regard to the participants’ gambling activities. Questions were asked to obtain information pertaining to the types, frequency and duration of gambling activities. Questions asked also included whom the participants typically gambled with, as well as how participants were first introduced to gambling. Questions also asked about their experiences with online transactions, the consequential impacts of online gambling and treatment barriers for compulsive online gambling-related difficulties. Lastly, questions were asked about the effectiveness of various harm minimization and responsible gambling measures.

The South Oaks Gambling Screen (SOGS) is a 20-item questionnaire that is based on the diagnostic criteria for pathological gambling, and it is one of the most commonly used screening tools for gambling disorders. It comprises questions ranging from the amount of money spent on gambling and even includes questions about whether others have perceived an individual to have a gambling problem. A score of 5 or above on the questionnaire is indicative of pathological gambling. The SOGS takes into consideration the gambling related issues in the past year. The SOGS has been previously evaluated in an Asian community in Singapore, and it was demonstrated to have high internal consistency with a Cronbach’s alpha coefficient of 0.84 [10].

The Hospital Anxiety and Depression Scale (HADS) is a 14-item scale which is commonly used to screen for potential anxiety and depression in participants. The scale consists of a series of statements that describe how respondents have felt over the course of a week. Individual items are rated on a 4-point scale indicating how often they have experienced the emotions described in the statements. Scores on the HADS are interpreted as follows: “0–7” are indicative of normal, “8–10” as mild, “11–14” as moderate and “15–21” as severe levels of anxiety and depression. Based on a prior review, the sensitivity and specificity of the HADS have been determined to be 0.80, and it has been found to be comparable to that of the General Health Questionnaire [11].

The Gambling Attitudes Scale is a 3-item questionnaire that assesses respondents’ perspectives towards the harm as compared to the benefits of gambling. It also assesses the morality of gambling and their opinion on the appropriate policy concerning the legalization of gambling.

### 2.6. Statistical Analysis

Questionnaire data was entered and coded into a de-identified SPSS database and subsequently analyzed using SPSS version 18 (SPSS Inc., Chicago, IL, USA) [12]. Statistical tests, which included Chi-square comparisons and Wilcoxon signed rank tests (for non-parametric comparisons) were utilized in the analysis of the dataset. Chi-square tests were used in the analysis of the dataset for demographic characteristics and online gambling characteristics. Wilcoxon signed rank tests were used to determine if there were significant differences in the amount of money bet.

## 3. Results

A cumulative total of 100 participants were recruited. Twelve percent of the participants had gambled solely online, and the remaining participants had engaged in both online and normal offline gambling. Table 1 illustrates the demographic characteristics of the sample. The vast majority of the participants were male, of Chinese ethnicity (91.0%) and under the age of 30 years old (48%). Most of the participants were single (58.0%) and had attained at least a pre-university level of education (51.0%). Eighty-nine percent of the participants were employed, and in terms of living arrangements, most were living with their family members (58.0%). Only 19% of the sampled participants gambled solely online, whereas the remaining participants engaged in both online and offline gambling. Chi-square analyses revealed that there were significant differences due to gender and marital status (χ^2^ = 9.043, *p* = 0.029) and familial/marital status (χ^2^ = 16.788, *p* = 0.001).

As mentioned previously, our sample mainly comprised participants who had sought help from our clinical services. Table 2 provides an overview of the scores on the respective questionnaires which were used to screen the participants. Ninety-eight percent of the sampled participants were characterized as pathological gamblers. The median score on the Hospital Anxiety and Depression Scale indicates that the vast majority of the participants self-reported mild levels of depressive and anxiety symptoms. In terms of genetic vulnerability, 20% of the participants reported another family member with gambling related problems.

Table 3 provides an overview of the online gambling characteristics reported by the participants sampled, as well as the consequential harm associated with online gambling. The vast majority (82.0%) were introduced to online gambling by their friends, and the most common reason provided for the initial attraction was the ease of access (85.0%). The vast majority (44.0%) reported that there was no specific time in the day that they gambled online, but at least 35% reported that they tend to gamble at night. Mobile phones and smartphone were the most commonly utilized platforms for gambling online. When questioned about the mechanisms by which bets were made online, most online gamblers reported that they utilize either bank transfer (55.0%) or transmission of cash involving a third party (53.0%). The vast majority of online gamblers bet on soccer (90.0%), followed by other sports (44.0) and casino games (27.0%).

Most participants reported that online gambling has affected them financially (97.0%), as well as through emotional problems (85.0%) and issues with their existing relationships (84.0%).

Table 4 provides an overview of the perceptions of the sampled participants with regard to the harm associated with gambling on society and the morality of gambling as well as the legalization of gambling. Whilst a good proportion of our sampled cohort had gambled online, 75.0% of them reported that the harm associated with gambling on society far outweighs the benefits. Notably, only 37.0% of the sampled participants felt that online gambling was definitely morally wrong. Thirty-nine percent of the sampled cohort perceived that some types of online gambling ought to be legalized. Of interest, 21.0% perceived that gambling should be legalized. The vast majority of the participants had concerns about being involved in fraud whilst gambling online. For each of the different perceptions and concerns, Chi-square analyses did not reveal any significant findings.

Table 5 compares the frequencies of gambling in terms of the various types of gambling activities between online and offline gamblers. Online gamblers tended to gamble almost daily on casino games, and weekly on lottery games, and weekly for horse and soccer betting. Whilst there were differences in the patterns of gambling between online and offline gamblers, further statistical analysis comparing the differences between the two groups by the type and frequency of gambling did not achieve any significant results.

However, there were significant differences found with regard to the amount of money bet. The median largest ever debt incurred as a result of online gambling ($20,000) was significantly more than that due to offline gambling ($500) (*Z* = −4.17, *p* < 0.001). As for the biggest ever loss, the median loss by participants was significantly larger from online gambling ($7000) (*Z* = −2.73, *p* < 0.01) compared to offline gambling ($2000). Similarly, the median amount for the biggest ever win on online gambling activities ($6000) was significantly greater than that for offline gambling activities ($3000) (*Z* = −3.07, *p* < 0.01). The median monthly amount for a win was also higher for online gambling activities, at $5000, whilst that for offline gambling was $1000 *(Z* = −6.15, *p* < 0.001).

Thirty-six participants also reported that they have engaged in ‘free-to-play’ games that simulate gambling. Most of these games were accessed through Facebook (47.2%) and by means of a smartphone application (55.6%). Table 6 provides an overview of the types of ‘free-to-play’ games engaged by participants. A total of 86.1% of the participants felt that these games were less addictive compared to online gambling. A total of 27.8% of the participants reported that virtual money encouraged them to gamble with real money.

Table 7 provides information that has resultant clinical implications, in that it looks at the duration since the first gambling experience as well as the barriers towards seeking treatment. In addition, it also provides an overview of some of the perceptions towards harm minimization measures or responsible gambling measures. A total of 18.4% of the participants had waited for between 1 and 2 years prior to seeking help for their gambling issues, but 17.3% of the participants had waited for more than 10 years prior to seeking help. With regards to the barriers leading to participants not seeking treatment, 20.8% of the participants reported that they have not considered help due to stigma, or they were pre-contemplative about their gambling issues, or they had the perception that treatment would be ineffective. This could have potentially accounted for the long latency prior to them seeking treatment.

Most participants perceived that self-exclusion was the most viable method by which excessive online gambling could be controlled. A good proportion of the participants also acknowledged that having penalties for online gambling would act as a deterrent.

## 4. Discussion

Our current study is perhaps one of the first studies in Singapore which has looked into the demographic profiles of online gamblers and has identified some of the harm associated with online gambling. In addition, the current study also highlights some of the perceptions towards harm minimization and responsible gambling measures, which have implications for policy planning. The demographic characteristics of our cohort of online gamblers is reflective of the treatment-seeking gamblers at the NAMS, Singapore [5], in that the clear majority of the gamblers were male, young, single, of Chinese ethnicity and had attained at least a tertiary level of education. The clear majority of our sampled participants were in service and sales or managerial roles, which corresponds to the findings of prior studies that have shown that online gamblers tend to be of higher socioeconomic status [13]. Prior studies have reported the consequential effects of online gambling on interpersonal and familial relationships, and this was evident in our current study as the clear majority of the participants were living with their immediate family members or partners [14,15].

Our current study showed that soccer betting was perhaps the most common type of online gambling activity amongst the sampled participants. This is congruent with prior findings by Fong & Ozorio [16] and Lee et al. [17], which highlighted that soccer betting was especially prevalent amongst the Chinese ethnicity group. Notably, our current study also highlights that there is a vast variety of online gambling activities that individuals engage in. The vast variety of games that individuals can engage in through online gambling could potentially increase the number of individuals seeking this modality of gambling due to the convenience and ease of access to a variety of gambling options. McCormack A. et al. [18] reported that the presence of multiple gambling options commonly serves as a motivator for individuals. The attractions of gambling online, such as the ease of access, privacy, ability to tap on electronic credits and better odds are some of the common çreasons that our participants reported to entice them to gamble online. This finding is congruent with what Gainsbury et al. [13] and Hing et al. [8] found. Our current study highlighted that there was a significant difference in the amount of money bet for online versus offline games. Griffiths & Parke [19] postulated that the increased bets whilst gambling online might be attributed to the fact that gambling using online credit impedes the sense of realism, thus leading to uncontrolled betting and greater financial consequences, given the intrinsic lower perceived value of electronic credits. We postulate that individuals are enticed towards online gambling as it offers more concealment and gamblers can anonymously gamble at any time in the privacy of their home, workplace, or even when they are outside on the go.

In the current study, we also looked at how monetary transactions take place for online gambling. Of note, most of the participants reported utilizing hand to hand monetary transactions and interbank transfers instead of credit card transactions. This is contrary to what is typically expected, in that credit card transactions ought to be more commonplace. We hypothesize that this might be because gamblers have a tendency not to use credit card transfers to avoid being tracked, also hypothesize that gamblers might have a false sense of security when their money is being transferred to overseas accounts, as they perceive that by doing so, they are able to evade law enforcers. In addition, we evaluated participants’ perceptions about ‘free-to-play’ games and virtual money. Notably, our participants reported that playing with virtual money in these ‘free-to-play’ games encouraged them to gamble with real money.

One of the main objectives of the current study was to determine online gamblers’ perceptions towards harm minimization strategies. Harm minimization for gambling disorders refers to strategies that aim to promote responsible gambling, whereby individuals gamble within their means, without spending more than the intended amount of time or money on gambling [20]. Prior studies have highlighted the efficacy of pop-up reminder messages to remind gamblers that they have spent a significant amount of time or money to alter their gambling pattern [21]. In our current study, this was, however, not the most effective measure perceived by online gamblers. Our sampled participants perceived self-exclusion measures, such as being able to exclude oneself from online gambling sites, as being more efficacious. While prior studies conducted overseas have advocated online self-exclusion as a viable harm minimization strategy [22,23], we are cognizant that in the local context, the involvement of family members in helping gamblers manage their online gambling habits might make this strategy more effective. This is similar to how the NCPG offers exclusion options for not only individuals themselves, but also for family members, from the local casinos. Whilst advances in technology might have led to an increased incidence of online gambling, technology could also be harnessed to control the amount that gamblers gamble. There is currently a myriad of gambling filtering products that could assist in preventing gamblers from accessing gambling websites.

There are several clinical implications that arise from the findings from our study. Given the incidence of online gambling in the Asian context, clinicians and addiction healthcare professionals need to further explore this new modality of gambling and how it has affected psychosocial functioning. It is important to recognize that online gambling is covert in nature. Hence, it is important that increased education is provided to family members, so that they are better able to identify the clinical signs and symptoms associated with online gambling and refer their loved ones to seek help early. Aside from enabling family members to recognize early clinical signs and symptoms of online gambling, it is also important to provide adequate support to family members, given that the findings from our current study highlighted that online gambling has a severe negative impact on family members. In addition, there needs to be a multi-pronged approach, involving not only treatment services, but also law enforcement, ministries and regulatory bodies, in order to collectively address the harm of online gambling.

There are several strengths of the current study. We managed to evaluate the online gambling habits and characteristics in a clinical population of gamblers. We also managed to evaluate the online gambling habits of Asian gamblers. There remains a paucity of literature about the online gambling habits of Asian gamblers, despite the increasing prevalence of the problem in this cohort. In addition, we also manage to gather online gamblers’ perceptions of various measures in which online gambling could be curtailed. We also managed to determine the average duration of time from their first gambling experience until they sought help from an addiction service, and we have also managed to identify the potential barriers that might hinder problem gamblers from seeking help. This can better inform and would potentially be of value and relevance for governmental policies that seek to deal with issues regarding online gambling.

Whilst there are several strengths of the current study, there remain several limitations which we need to acknowledge. In terms of research design, our current study is a cross-sectional study and it would be ideal for this to be a cohort study to determine longitudinal progression and outcomes. The sampled cohort of participants might not be representative of the general population, given that they are a cohort of participants who have decided to seek help from an addiction service. In addition, we have not made use of validated questionnaires to determine the characteristics of the online gambling problems and consequential harm. Due to the lack of follow-up as this is a cross-sectional study, we are unable to determine the longer-term prognosis.

## 5. Conclusions

This is perhaps one of the first Asian studies to look into the serious harm involved in online gambling. The findings from our study can guide further interventions in the treatment of online gambling related disorders and could be of interest to governmental organizations in their planning of regulations for online gambling.

## Figures and Tables

**Table 1:** Demographic characteristics of the sampled population (n = 100).

Demographics		Number	*p*-Value
Gender	Male	98	NA
	Female	2	
Age	21–30	48	0.904
	31–40	33	
	41–50	14	
	51–60	5	
	Mean	32.9	
Race	Chinese	91	0.977
	Indian	6	
	Malay	2	
	Others	1	
Marital Status	Single	58	0.029
	Married	36	
	Divorced	5	
	Widowed	1	
	University degree and above	22	0.654
	ITE (Institute of Technical Training)/Diploma/Pre-University	51	
	Secondary	27	
	Mean years of formal education	12.9	
Religion	Christianity	22	0.965
	Buddhism	36	
	Taoism	6	
	Hinduism	2	
	Islam	2	
	Others	32	
Employed	Yes	89	0.075
	No	11	
Occupation type (coded via Singapore Standard Occupational Classification 2015)	Unemployed	11	0.651
	Service and sales workers	32	
	Professionals	22	
	Legislators, senior officials and managers	15	
	Armed forces personnel	12	
	Associate professionals and technicians	7	
	Craftsmen and Related Trades Workers	1	
Living status	Living with family of origin (parents/siblings)	58	0.001
	Living with spouse/children	28	
	Living alone	3	
	Others	11	

**Table 2:** Scores on the South Oaks Gambling Screen (SOGS) and Hospital Anxiety and Depression Scale (HADS) for sampled participants and genetic predisposition.

Questionnaires		Score	Percentage/Remarks
South Oaks Gambling Screen (SOGS) (0: no problems; 1–4: some gambling problems; 5 or more: pathological gambler)	Minimum	1	(Some gambling problems)
	Maximum	19	(Pathological gambler)
	Median	12.2	(Pathological gambler)
	Mean	12.0	(Pathological gambler)
Hospital Anxiety and Depression Scale (HADS)—Depression (0–7: normal; 8–10: mild; 11–14: moderate; 15–21: severe)	Minimum	0	(Normal)
	Maximum	21	(Severe)
	Median	8.0	(Mild)
	Mean	8.2	(Mild)
Hospital Anxiety and Depression Scale (HADS)—Anxiety (0–7: normal; 8–10: mild; 11–14: moderate; 15–21: severe)	Minimum	0	(Normal)
	Maximum	21	(Severe)
	Median	9.0	(Mild)
	Mean	8.5	(Mild)
Family members or acquaintances with gambling problems	Parent(s)	20	20.0%
	Children	0	0.0%
	Sibling(s)	6	6.0%
	Other Relatives	14	14.0%
	Spouse	0	0.0%
	Friend(s)	33	33.0%

**Table 3:** Online gambling characteristics.

Gambling Characteristics		Percentages (%)
Platform of introduction to online gambling	Friends	82.0
	Internet (website)	19.1
	Colleagues	15.7
	Social Media	12.4
	Family/Relatives	4.5
	Others	5.6
Reasons for initial attraction to online gambling	24-h access	85.0
	Convenience/Privacy	68.0
	Higher returns than offline gambling	57.0
	Variety of games	48.0
	Electronic cash	47.0
	No entry levy	46.0
	Absence of monitoring	30.0
	Ease of access to additional credit facilities	15.0
Time of the day preference for gambling online	No specific time	44.0
	Only at night	35.0
	After work	21.0
	During the day	5.0
Platforms of choice for gambling online	Mobile phone	88.0
	Home Computer	56.0
	Tablet/iPad	21.0
	Computer at workplace	12.0
Mechanism of payment for bets made online	Bank transfer	55.0
	Transmission of cash involving a third party	53.0
	Credit card	10.0
	E-Wallets	14.0
	Others	6.0
Reasons for preference for online gambling	Convenience	47.0
	Choice of credit payment	14.0
	Attractive odds	12.0
	Variety of games	9.0
	Privacy	9.0
	Live betting	1.0
Types of online gambling activities engaged in	Soccer betting	90.0
	Other sports betting	44.0
	Casino games	27.0
	Lottery	17.0
	Poker	13.0
	Horse racing	5.0
	Others	8.0
Country of origin of websites that participants gamble on	Southeast Asia	25.0
	United Kingdom	17.0
	USA	5.0
	Asia	5.0
Most problematic online gambling activities	Soccer betting	69.0
	Casino games	20.0
	Other sport betting	15.0
	Poker	3.0
	Lottery	3.0
	Horse racing	3.0
	Others	2.0
Areas of impact of online gambling on a participant‘s life	Finances	97.0
	Emotional problems	85.0
	Relationships	84.0
	Time	79.0
	Stress	78.0
	Concentration	74.0
	Sleep	77.0
	Fatigue	59.0
	Appetite	48.0
	Health	40.0
Addictive or problematic features of online gambling activities	Accessibility and convenience	28.0
	Attractive odds	15.0
	Live betting	14.0
	No upfront cash needed	13.0
	Thrill from winning money	12.0
	Variety of games	9.0
	Ease of betting	7.0
	Fast-paced game plot	6.0
	More betting options	5.0
	Immediate returns	4.0
	No limits to betting amount	2.0

**Table 4:** Perceived harm and concerns pertaining to online gambling.

Perceptions and Concerns		Percentages (%)
Perception of harm associated with gambling on society	Harm definitely outweighs the benefits	75.0
	Harm somewhat outweighs the benefits	13.0
	Harm is equal to the benefits	8.0
	Benefits outweigh the harm	4.0
	Benefit far outweighs the harm	0.0
Morality associated with gambling	Not considered morally wrong	41.0
	Definitely morally wrong	37.0
	Neither morally right nor wrong	22.0
Perceptions towards the legalization of gambling	All forms of gambling should be legalized	21.0
	Some types should be legal, and other types illegal	39.0
	All types of gambling should be illegal	13.0
	Unsure or do not know	27.0
Concerns about gambling online	Fraud	42.0
	Legality	40.0
	Security of financial transactions	28.0
	Lack of player protection measures	24.0
	Lack of regulation	19.0

**Table 5:** Differences between online and offline gamblers by modality of gambling activities.

Type of Gambling Activity	Frequency of Gambling	Offline Gambling (%)	Online Gambling (%)
Casino gambling	Daily	1.9	17.0
	Almost daily	7.5	9.4
	Weekly	18.9	9.4
	Fortnightly	1.9	1.9
	Monthly	15.1	3.8
	More than monthly	32.1	5.7
Lottery games	Daily	1.50	1.50
	Almost daily	12.10	7.60
	Weekly	45.5	12.10
	Fortnightly	12.10	1.50
	Monthly	19.70	1.50
	More than monthly	1.50	1.50
Poker card games (non-casino)	Daily	0	11.5
	Almost daily	15.4	7.7
	Weekly	11.5	11.5
	Fortnightly	7.7	7.7
	Monthly	26.9	11.5
	More than monthly	15.4	0
Horse betting	Daily	0	20.0
	Almost daily	20.0	10.0
	Weekly	50.0	20.0
	Fortnightly	0	0
	Monthly	0	0
	More than monthly	0	0
Soccer betting	Daily	5.40	47.80
	Almost daily	8.70	26.10
	Weekly	8.70	15.20
	Fortnightly	2.20	2.20
	Monthly	8.70	3.30
	More than monthly	5.40	2.20
Transactions/amount of money bet	Largest median debt	500	20,000
	Biggest median loss	2000	7000
	Biggest median win	3000	6000
	Monthly median win	1000	5000

**Table 6:** Types of free-to-play games engaged in by participants.

Types of ‘Free-to-Play’ Games Engaged in Poker	66.7%
Casino	19.4
Slots	16.6
Mahjong	16.6
Jackpot	8.3

**Table 7:** Treatment barriers and perceptions towards harm minimization measures.

Treatment Barriers and Perceptions towards Harm Minimization		%
Perceived barriers against seeking treatment (%)	<1 year	10.2
	1–2 years	18.4
	2–3 years	6.1
	3–4 years	9.2
	4–5 years	5.1
	5–6 years	5.1
	6–7 years	6.1
	7–8 years	6.1
	8–9 years	3.1
	9–10 years	13.3
	More than 10 years	17.3
Barriers to seeking treatment (%)	Stigma	20.8
	Does not perceive gambling to be a problem	20.8
	Treatment would not be effective	20.8
	No indication for treatment	14.6
	Lack of time	14.6
	Cost due to current debts	12.5
	Location/geographical barriers	12.5
	Confidentiality issues	4.2
Perceived effectiveness of responsible gambling measures in minimizing excessive or pathological gambling (%)	Self-exclusion via online website	3.76
	Pre-commitment (limiting spending to a fixed amount per day)	3.07
	Responsible gambling tool	2.69
	Prominent display of responsible gambling messages on where to seek help	2.39
	Pop-up messages summarizing history of deposit and play activities	2.08
	Compulsory breaks in play	1.60
Perceptions about having penalties towards gambling online (%)	Strongly disagree	6.0
	Disagree	8.0
	Neutral	25.0
	Agree	39.0
	Strongly agree	22.0
Beliefs about continued illegal online gambling activity even amidst legal alternatives (%)	Strongly disagree	19.0
	Disagree	25.0
	Neutral	25.0
	Agree	21.0
	Strongly agree	10.0
